# Outcomes of prostatic urethral lift in a medically complex population at a veterans affairs hospital

**DOI:** 10.1002/bco2.126

**Published:** 2021-12-04

**Authors:** Kelly Lehner, Shreeya Popat, Katherine Utech, Jennifer Taylor, Michael Brooks, Jeffrey Jones

**Affiliations:** ^1^ Scott Department of Urology Baylor College of Medicine Houston Texas USA; ^2^ Department of Urology Michael E. DeBakey Veteran Affairs Medical Center Houston Texas USA

**Keywords:** BPH, prostatic urethral lift, urolift, veterans

## Abstract

**Objective:**

The objective of this study is to report “real‐world” outcomes of prostatic urethral lift (PUL) in a medically complex US military veteran population while employing liberalized procedural indications.

**Methods:**

A retrospective review was conducted of patients who underwent PUL at our institution. There were no prostatic size requirements, patients were accepted on anti‐platelet/anticoagulant therapy, no benign prostatic hyperplasia (BPH) medication washout was required, and there was no maximum post‐void residual PVR. Pre‐ and post‐operative International Prostate Symptom Score (IPSS), uroflowmetry, and PVR were recorded. Statistical comparisons were performed using simple *t* tests.

**Results:**

From 2013 to 2019, 91 patients underwent PUL. Mean age was 70 (range 55–92) years. The majority of our patients were classified as American Society of Anesthesiologists (ASA) class 3 versus the general population at ASA class 2. Post‐operatively, IPSS decreased by an average of 43% (23 to 13, *p* < 0.001). There was a mean 41% decrease in PVR (179 to 101 cc, *p* = 0.009), which was durable for a follow‐up of up to 54 months. Maximum urinary flow rate improved by an average of 32% (9.3 to 12.3 cc/s, *p* = 0.003), which was also durable throughout follow‐ up. Forty‐four patients required catheterization pre‐operatively and 16 required catheterization post‐operatively. Therefore, 27 patients (61.4%) were rendered catheter‐free by PUL. Thirty‐nine patients were taking antiplatelet medications peri‐operatively, and 13 took anticoagulants. Only one patient (on warfarin) experienced hematuria requiring re‐admission with catheter placement.

**Conclusions:**

PUL produced effective and durable results in our veteran population, including in patients with significant pre‐operative bladder decompensation and those on antiplatelets/anticoagulants.

## 1 INTRODUCTION

Prostatic urethral lift (PUL) is a minimally invasive intervention for symptomatic benign prostatic hyperplasia (BPH) that has been approved for use in the United States since 2013 for prostate sizes up to 80 g.^1^ In PUL, a rigid cystoscope is used to place transprostatic clips onto the anterolateral prostate, compressing prostatic tissue to create an open anterior lumen.^2^ For patients who do not receive adequate symptom relief from medical management of BPH or for those who find medication side effects to be intolerable, PUL has emerged as an alternative to prostatic vaporization or traditional transurethral resection of the prostate (TURP).^3^

While studies have shown that PUL is effective in improving lower urinary tract symptoms (LUTS), more work is needed to establish the efficacy of PUL in a real‐world clinical setting, with liberalized inclusion and exclusion criteria applied.^4^, ^5^ In addition, previous work has not reported use of PUL in populations with significant medical comorbidities, which are associated with increased operative risk and higher frequency of adverse outcomes in procedures, even minimally invasive ones. This study examines outcomes of PUL in a medically complex US military veteran population, while employing more liberalized patient selection criteria.

## 2 BACKGROUND

PUL may present several advantages over TURP in reducing side effects from the procedure, while having acceptable improvements in objective voiding measures. PUL has been shown across multiple studies to provide effective relief for LUTS from BPH.^6^ While the international prostatic symptom score (IPSS) improves by 3–5 points with medical management, IPSS improves by up to 11 points with PUL, compared to 14 points with TURP.^1^ PUL has additionally performed better than TURP using an alternative characterization of BPH symptoms called the BPH6, which includes specific endpoints for LUTS relief, recovery experience, erectile function, ejaculatory function, continence preservation, and safety. In a prospective randomized controlled trial of 80 patients across 10 European centers, PUL was found to have better outcomes in the BPH6 domains than TURP overall, with 52.3% of individuals who underwent PUL meeting BPH6 primary endpoints versus 20.0% of those who underwent TURP (noninferiority *p* < 0.0001; superiority *p* = 0.005).^7^, ^8^

While TURP carries a small risk of post‐operative erectile dysfunction (ED), studies have demonstrated that PUL may actually even improve the Sexual Health Inventory in Men (SHIM) score in men with severe erectile dysfunction (ED).^9^ In addition, PUL has also been found to have cost‐savings associated with its ability to be performed as an outpatient procedure and its low complication rates.^10^, ^11^ Modeling the UK National Institute for Health and Care Excellence showed that when PUL is performed as an outpatient surgery, PUL produced savings of £286 per patient compared to monopolar TURP £159 per patient compared to bipolar TURP and £90 per patient compared to holmium laser enucleation of the prostate (HoLEP).^11^

The efficacy of PUL was initially demonstrated in a clinical trial with 5‐year follow‐up, in the Luminal Improvement Following Prostatic Tissue Approximation (LIFT) study by Roehrborn et al.^12^, ^13^, ^14^, ^15^ This sham‐controlled trial with 206 subjects showed IPSS improvement of 41.1% at 3 years and 36% at 5 years. The study group faced fairly rigorous inclusion criteria, including no prior surgical treatment for BPH, 2‐week *α*‐blocker washout, 3‐month 5*α*‐reductase inhibitor washout, maximum urinary flow rate (Qmax) of 12 ml per second or less, 30 to 80 cc prostate, no median lobe obstruction, and pre‐operative post‐void residual (PVR) urine volume less than 250 ml.^14^

Despite multiple studies that have proven the broad possible applications of PUL, we aim to enhance the current literature by showing that PUL is likewise effective in patients with significant medical comorbidities (including many on long term anti‐platelet or anti‐coagulant therapy) and in patients with considerable pre‐operative bladder deconditioning and urinary retention.

## 3 METHODS

This study was approved by the Institutional Review Boards at Baylor College of Medicine and the Michael E. DeBakey VA Medical Center (VAMC) in Houston. The charts of patients who underwent PUL at this large, tertiary care VAMC from 2013 to 2019 were reviewed retrospectively. Clinical characteristics of patients, including demographics, presence of urinary retention, use of anticoagulant/antiplatelet agents, and medical comorbidities, including ASA score, were recorded. Pre‐ and post‐operative subjective and objective parameters, such as International Prostate, or American Urological Association, Symptom Scores (IPSS or AUASS), Sexual Health Inventory in Men (SHIM) score, flow rate, and post‐void residual (PVR) bladder scan, were collected. In addition, details of operative procedures were noted. Pre‐ and post‐operative parameters were compared using paired *t* tests. Post‐operative complications and need for additional prostate procedures were also recorded and reported descriptively.

The inclusion criteria were men with symptomatic BPH who had failed medical therapy or who desired surgical intervention for their BPH symptoms who were surgical candidates. Our local VA urology practice removed urinary retention requiring catheter drainage as an exclusion criterion for PUL, as a significant number of veterans were referred to urology in retention yet were observed to have robust bladder contractility on pressure‐flow studies. These patients often had a high comorbidity index, so we aimed to study if this surgical modality could be used to relieve their outlet obstruction effectively.

## 4 RESULTS

From 2013 to 2019, 91 veteran patients underwent PUL at our institution (Table 1). Diagnosis of BPH with prostate suitable for PUL included IPSS, uroflowmetry, and PVR, followed by additional assessment pre‐operatively to include prostate specific antigen (PSA), cystoscopy, and transrectal ultrasound (TRUS). The vast majority of the patient population was on medical therapy for BPH pre‐procedurally (97.8%), while three individuals (3.3%) had prior surgical treatment for BPH by TURP. Median follow‐up time was 18 months, with maximum follow‐up of 54 months. First follow‐up visit was conducted on average at 30 days. The average patient age was 70 (range 55–92 years). Of those procedures, 53 were performed under general anesthesia, 35 with intravenous sedation and intravesical plus urethral lidocaine, and 3 under spinal anesthesia. Average American Society of Anesthesiologists (ASA) classification was 3.14. The average number of implants placed was 5 (range 2–13). Pre‐operative prostate size, as measured on TRUS, ranged from 14 to 115 cc, with an average size of 40 cc. Three patients had prostate volumes larger than 80 cc. A large median lope was identified by cystoscopy at the time of the PUL procedure, which was seen in 11 patients (12.1%).

**TABLE 1:** Patient demographics

Characteristic	*N* = 91
Age (year)	
Mean ± SD	70 ± 8.1
Range	55–92
Prostate Volume (cc)	
Mean ± SD	40.3 ± 18.5
Range	14–115
Operation Time (min)	
Mean ± SD	47.1 ± 31.3
Range	9–221
Implant Count per Patient	
Mean ± SD	5.2 ± 2.2
Range	2–13
ASA Class^a^	
Mean ± SD	3.1 ± 6.5
Type of Anesthesia, n	
General	53 (58.2%)
Local	35 (38.5%)
Spinal	3 (3.3%)
Scheduled Anticoagulation, n	45 (49.5%)
Pharmaceutical Treatment of LUTS, n	89 (97.8%)

Abbreviations: cc, cubic centimeter; LUTS, lower urinary tract symptoms; min, minutes; SD, standard deviation.

^a^ ASA, American Society of Anesthesiologists Classification for risk‐stratifying patients undergoing general anesthesia and ranges with increasing severity from 1 to 6.

Subjectively, IPSS decreased significantly (23 to 13, *p* < 0.001) by a mean of 10 points, consistent with prior studies. The average SHIM score changed from 8.1 pre‐operatively to 9.1 post‐operatively, a trend towards improved erectile function that was not statistically significant (*p* = 0.57). Objectively, there was a significant decrease in post‐void residual urine volumes after PUL (179 to 101 cc, *p* = 0.009), which was found to be durable for a follow‐up of up to 54 months (Table 2). In addition, the peak urinary flow rate improved significantly (9.3 to 12.5 cc/s, *p* = 0.003), which was also found to be durable for a follow‐up of up to 54 months.

**TABLE 2:** Quantitative measurements of outcomes

Outcome	Pre‐Op	Post‐Op
Catheter Status		
Data Reported	90	89
Indwelling	26 (28.9%)	13 (14.6%)
Change		−13 (−50%)
CIC	18 (20%)	6 (6.7%)
Change		−12 (−66.6%)
PVR (ml)		
*n*	76	83
Mean ± SD	179.4 ± 181.9	101.4 ± 116.2
*p* value		0.0014
Change		−78 (−43.5%)
IPSS		
n	57	56
Mean ± SD	23.3 ± 6.6	13.3 ± 6.9
*p* value		< 0.00001
Change		−10 (−42.9%)
SHIM		
*n*	34	32
Mean ± SD	8.1 ± 6.5	9.1 ± 7.3
*p* value		0.5699
Change		1.0 (12.3%)
Qmax (ml/s)		
*n*	60	54
Mean ± SD	9.3 ± 5.4	12.5 ± 6.8
*p* value		0.0062
Change		3.2 (34.4%)
Qavg (ml/s)		
*n*	47	39
Mean ± SD	5.2 ± 3.0	6.1 ± 3.1
*p* value		0.1728
Change		1.1 (21.2%)

The surgical failure rate of PUL in our patient population was 11.0%. Two individuals in our study (2.1%) underwent a second PUL procedure, and eight individuals (8.8%) underwent subsequent TURP for persistent bothersome symptoms after initial PUL. There was no significant difference (*p* > 0.05) in the median age, pre‐operative PVR, IPSS, Qmax, or prostate volume between individuals who did and did not require a second prostate procedure. All men on medical therapy were continued on medical therapy post‐operatively to provide some benefit in post‐operative edema, bleeding, and irritative symptoms. Via shared‐decision making with the patient and their primary care provider, medications were tapered as the flow rate, PVR, and LUTS allowed.

Concurrent bipolar “button” plasma vaporization of the prostate, while undergoing the PUL procedure, was used on a high bladder neck in 6 individuals (6.6%) and on a large median lobe in 11 individuals (12.1%), prior to demonstration and approval of use of the PUL on the median lobe. This operative plan was typically developed via shared‐decision making between the patient and a surgeon, while a pre‐operative conference of staff urologists reviewed and approved all operative plans. The operative strategies were devised to minimize operative times and bleeding risks (as opposed to the traditional TURP) in this medically complex population, while still hopefully maximizing improvement in LUTS. Overall operative time ranged from 9 to 221 min, with longer procedure times generally accounted for by coupling with additional procedures, such button TURP, bladder stone removal, or urethrotomy.

Of our 91 patients, 44 had complete urinary retention pre‐operatively: 18 (20%) were performing clean intermittent catheterization (CIC), and 26 (28.9%) had indwelling Foley catheters (Table 2). Post‐operatively, these numbers had reduced to 6 (6.7%) patients performing CIC and 13 (14.6%) with indwelling Foley catheters. Thus, 27/44 (61.4%) of catheter‐dependent patients were rendered catheter‐free after PUL.

In our population, 45 patients were taking antiplatelet or anticoagulant medications peri‐operatively, and 17 had these medications held prior to surgery. Of these patients, only one patient taking warfarin had hematuria postoperatively that required re‐admission but not re‐operation. Of note, this patient had undergone concurrent “button” TURP of the median lobe. Regarding other post‐operative complaints or complications, 14 patients reported dysuria (15.4%), 14 reported hematuria (15.4%), and 5 reported pelvic pain (5.5%). Eight individuals (8.9%) experienced urinary tract infections (UTI). Post‐operative erectile dysfunction was reported by only one individual (1.1%). Nine individuals returned to the emergency room within 90 days of their procedure: four for UTI, two for hematuria, and three for acute retention (Table 4).

One individual in our study had a prior history of prostate cancer treated with radiation therapy. No fibrosis, atrophy, or scarring was noted intraoperatively, and no difficulty with placement of clips occurred. Three individuals (3.3%) had undergone a prior TURP and requested minimally invasive reoperation, which similarly did not cause any operative difficulties or complications. Three individuals (3.3%) went on to develop prostate cancer, but none underwent prostatectomy so we are unable to report how prior PUL may have affected surgical extirpation in those cases.

## 5 DISCUSSION

In this study, we report our experience with PUL using more liberalized inclusion criteria, in a medically complex US military veteran population. Prior studies examined PUL within a more selected patient population with tight inclusion/exclusion criteria. However, there remains a need to determine if PUL can achieve satisfactory results when implemented with wider latitude in a real‐world clinical setting among more medically complex patients.

Specifically, the LIFT trial excluded patients with the following factors that we included in our surgical population: PVR > 250 ml, IPSS < 13, Qmax > 13 ml/s, prostate volume > 80 cc, obstructive or protruding median lobe of the prostate, previous BPH procedure, previous pelvic surgery or irradiation, history of prostate or bladder cancer, *α*‐blocker within 2 weeks of procedure, or 5*α*‐reductase inhibitor within 3 months of procedure. In our study population, 22.4% of patients had PVR > 250 ml, 20% had Qmax > 13 ml/s, 3.7% had prostate volume > 80 cc, 15.4% had an enlarged median lobe, 6.6% had a history of prostate or bladder cancer, and 3.7% had prior TURP. In addition, 97.8% of individuals were on pharmacotherapy for BPH, which was continued until the date of surgery. In many ways, our patient population was not selected or restricted to maximize the apparent outcomes for PUL, yet the results achieved are comparable to that seen in the LIFT trial. At 1 year, the LIFT trial showed a 10.61‐point reduction in IPSS and a 4.03 ml/s increase in Qmax. PVR and SHIM did not change significantly.^15^ By comparison, our study showed a 10.0‐point reduction in IPSS and a 3.2 ml/s increase in Qmax. Our data on change in PVR demonstrated a statistically significant decrease from 179.4 ± 181.9 to 101.4 ± 116.2. In addition, the total percentage of our patient population requiring catheterization (indwelling or clean intermittent catherization) decreased from 48.9% pre‐operatively to 21.3% post‐operatively.

Sievert et al.^16^ reported an investigation of PUL outcomes with obstructive median lobe as the only exclusion criteria in a cohort of 86 German patients. Unlike previous studies, they included patients with PVR greater than 250 ml, recurrent prostate‐related hematuria, and prostate volume greater than 60 ml. While their work provided valuable information that PUL can achieve efficacy similar to that reported in the LIFT trial with a wider range of pre‐operative metrics, their pre‐operative characteristics are still more favorable than what is observed in a typical elderly American veteran population. The German cohort had a pre‐operative indwelling catheter rate of 16.3%, as compared to 28.9% of our study population. An additional 20% of our study participants required CIC. Sievert et al.^16^ also report 8.1% of patients having a PVR greater than 250 ml, which was less than half of the incidence in our study population. In addition, the average age of patients in the German cohort was 66.2 ± 11.5, while it was 70 ± 8.1 in our study population. These values suggest a significantly higher level of bladder decompensation and retention in the patients we describe, which provides important support that PUL can be effective even in these circumstances.

Eure et al.^17^ additionally report real‐world outcomes of PUL in their multi‐center trial including 1413 patients across 14 institutions in the United States and Australia. Despite their more liberalized inclusion criteria than the original L.I.F.T. trial, patients in our study were different than theirs in several important ways. First, our patients were more symptomatic at baseline, with average pre‐procedural IPSS of 23 ± 6.6 as compared to 19.1 ± 7.0 and Qmax of 9.3 ± 5.4 as compared to 13.9 ± 10.2. In addition, although they included patients in urinary retention in their study, out study population included 48% of patients in urinary retention pre‐procedurally as compared to 11.7% pre‐procedurally in their study. Finally, their study does not report on the prevalence of significant medical comorbidities or ASA score. Despite the differences in patient population, our studies report similar improvements in IPSS and Qmax outcomes. We show a 10‐point reduction in IPSS in our study versus an 8.1‐point reduction in IPSS in their study. We demonstrate a 3.2 ml/s improvement in Qmax, while they demonstrate a 0.4 ml/s improvement in Qmax. Our study furthers their conclusion that PUL is highly effective in a real‐world non‐selected patient population by showing that even with high levels of pre‐operative retention and significant medical comorbidities, the procedure remains safe, effective, and durable.

We describe the successful implementation of PUL in a highly comorbid population. The average ASA classification among our patient cohort was 3.14. An ASA score of 3 is defined as “patients with severe systemic disease,” while 4 is defined as “patients with severe systemic disease that is a constant threat to life.” The US population has an average ASA class of 2, and an age‐matched general genitourinary patient population was shown to have an ASA of 2.5, showing that veterans have an age‐for‐age higher comorbidity rate than the civilian population.^18^ Of the patients included in our study, 26.4% had coronary artery disease, 15.4% had chronic obstructive pulmonary disease (COPD), 71.4% had hypertension, and 40.7% had diabetes (Table 3). PUL is a suitable procedure in patients with high levels of surgical risk because it can be well tolerated under local anesthesia ± sedation, with low levels of discomfort or pain.^19^ Even so, 58.2% of our PULs were conducted under general anesthesia with no adverse events or intraoperative complications noted. The average total procedure time was 59 ± 37 min with general anesthesia and 35 ± 15 min with local anesthesia. The procedures were conducted in the operating room at our VA hospital because the outpatient clinic procedure rooms are not approved for conscious sedation.

**TABLE 3:** Summary of comorbidities of study population

	Subjects, *n*		Subjects, *n*
Genitourinary	47 (51.6%)	Non‐genitourinary	86 (94.5%)
Impotence	13 (14.3%)	OSA	13 (14.3%)
Renal mass/cancer	5 (5.5%)	Diabetes mellitus	37 (40.7%)
Bladder mass/cancer	4 (4.4%)	Hypertension	65 (71.4%)
Testicular cancer	1 (1.1%)	Hyperlipidemia	55 (60.4%)
Prostate cancer	2 (2.2%)	Atrial fibrillation	8 (8.8%)
Hydrocele	2 (2.2%)	Cerebrovascular accident	9 (9.9%)
Prostatitis	1 (1.1%)	COPD	14 (15.4%)
Balanitis	1 (1.1%)	GERD	19 (20.9%)
Recurrent UTIs	8 (8.8%)	CKD	10 (11.0%)
Calculi	9 (9.9%)	Obesity	15 (16.5%)
Renal transplant	3 (3.3%)	Peripheral vascular disease	4 (4.4%)
Obstructive uropathy	2 (2.2%)	Congestive heart failure	9 (9.9%)
		Coronary artery disease	24 (26.4%)

Abbreviations: CKD, chronic kidney disease; COPD, chronic obstructive pulmonary disease; GERD, gastroesophageal reflux disease; OSA, obstructive sleep apnea.

The rate of reported surgical complications from PUL varies widely across studies. The most commonly reported complication is hematuria, which has been shown to range from 4% to 78% of patients undergoing PUL. Dysuria is reported in 9% to 73% of patients, pelvic pain in 6% to 52% of patients, and urinary tract infection (UTI) in 0.1% to 7%.^3^, ^8^, ^13^, ^19^, ^20^ Despite a medically complex patient population, with many on anti‐coagulants and other medications, our complication rate fell inside these bounds (Table 4), with hematuria in 14/91 (15.4%) of patients, dysuria in 15/91 (16.5%), and pelvic pain in 5/91 (5.5%). Post‐operative UTI developed in 8/91 (8.8%). The baseline level of erectile dysfunction in our population was high, with a pre‐operative average SHIM score of 8.1, representing moderate ED. The average SHIM score was 9.1 post‐operatively, a trend towards improved erectile function that was not statistically significant. Prior studies have similarly shown SHIM score to be largely unaffected by PUL.^7^, ^9^, ^20^

**TABLE 4:** Post‐operative adverse events

Event	Subjects, *n*
Hematuria	14 (15.4%)
Dysuria	15 (16.5%)
Infection	8 (8.8%)
Erectile dysfunction	1 (1.1%)
Retrograde ejaculation	0 (0%)
Pain	5 (5.5%)
Emergency department visit within 90 days	9 (9.9%)
UTI	4 (4.4%)
Hematuria	2 (2.2%)
Acute retention	3 (3.3%)

The durability of our results is also comparable to that seen in the LIFT trial and the BPH6 trial. The BPH6 trial had a 13.6% 2‐year retreatment rate, and the LIFT study likewise had a 13.6% 5‐year retreatment rate (4.3% receiving additional PUL implants and 9.3% receiving TURP or laser ablation).^21^ In our study, 2.2% of individuals went on to receive additional PUL implants, and 8.8% went on to receive a TURP for an overall retreatment rate of 11.0% throughout follow‐up of up to 4.5 years.

The limitations of our paper include its retrospective nature and the lack of a sham control group to assess comparative outcomes. In addition, our patients did not routinely undergo urodynamic study (UDS) to assess for other etiologies of voiding dysfunction, which may in part explain why 19% still required CIC or indwelling Foley following PUL. Our experience suggests that in patients where bladder decompensation is suspected, clinicians may be advised to routinely perform UDS prior to consideration of procedures to address BPH. Finally, our paper was limited by the fact that PUL was performed by several different clinicians at the attending and resident level, meaning we were unable to control for specific surgeon technique or level of experience.

## 6 CONCLUSIONS

Our real‐world clinical application of the PUL demonstrated that it has effective, safe, and durable results in a medically complex veteran population, including in patients requiring catheterization, patients with significant urinary retention, and those on antiplatelets/anticoagulants. Based on our findings, we conclude that PUL is an appropriate treatment option for symptomatic BPH, even in individuals with severe LUTS and significant medical comorbidities.

## CONFLICT OF INTEREST

The authors declare that they have no affiliations with or involvement in any organization or entity with any financial interest or non‐financial interest in the subject matter or materials discussed in this manuscript.
